# Interval appendectomy practices for complicated appendicitis in children: a systematic review from the APSA Outcomes and Evidence-Based Practice Committee

**DOI:** 10.1007/s00383-026-06445-z

**Published:** 2026-04-27

**Authors:** Jason P. Sulkowski, Carlos T. Huerta, Jun Tashiro, Diana L. Diesen, Brian C. Gulack, Emily Christison-Lagay, Katie W. Russell, Hanna Alemayehu, Stephanie F. Polites, Matthew T. Hey, Henry L. Chang, Alana L. Beres, Romeo C. Ignacio, Donald J. Lucas, Sandra K. Kabagambe, Robert Baird, Afif N. Kulaylat, Sara A. Mansfield, Rebecca M. Rentea, Christopher Pennell, Barrie S. Rich, Yasmine Yousef, Robert Ricca, Lorraine Kelley-Quon, Tamar L. Levene

**Affiliations:** 1 Division of Pediatric Surgery, Comer Children’s Hospital, University of Chicago, 5841 S. Maryland Ave, Rm A-415 MC 4062, Chicago, IL 60637 USA; 2 DeWitt Daughtry Family Department of Surgery, Division of Pediatric Surgery, University of Miami, Miami, FL USA; 3 Division of Pediatric Surgery, Department of Surgery, New York University Grossman School of Medicine, New York, NY USA; 4 Department of Surgery, Division of Pediatric Surgery, University of Texas Southwestern, Dallas, TX USA; 5 Department of Surgery, Rush University Medical Center, Chicago, IL USA; 6 Department of Surgery, Division of Pediatric Surgery, Yale School of Medicine, New Haven, CT USA; 7 Division of Pediatric Surgery, University of Utah, Salt Lake City, UT USA; 8 Division of Pediatric Surgery, Emory University School of Medicine, Children’s Healthcare of Atlanta, Atlanta, USA; 9 Division of Pediatric Surgery, Mayo Clinic, Rochester, MN USA; 10 Department of Surgery, Brigham and Women’s Hospital, Boston, MA USA; 11 Department of Pediatric Surgery, Johns Hopkins All Children’s Hospital, St. Petersburg, FL USA; 12 Division of Pediatric General and Thoracic Surgery, St. Christopher’s Hospital for Children, Drexel University College of Medicine, Philadelphia, PA USA; 13 Division of Pediatric Surgery, University of California San Diego School of Medicine, La Jolla, CA USA; 14 Division of Pediatric Surgery, Children’s Hospital Los Angeles, University of Southern California, Los Angeles, CA USA; 15 Carle Foundation Hospital, Carle Illinois college of Medicine, Urbana-Champain, IL USA; 16 Division of Pediatric Surgery, BC Children’s Hospital, Vancouver, BC Canada; 17 Division of Pediatric Surgery, Penn State Children’s Hospital, Hershey, PA USA; 18 Division of Pediatric Surgery, Nationwide Children’s Hospital, Columbus, OH USA; 19 Division of Pediatric Surgery, Children’s Mercy-Kansas City, University of Missouri- Kansas City, Kansas City, MO USA; 20 Division of Pediatric Surgery, Mercy Hospital, Saint Louis, MO USA; 21 Division of Pediatric Surgery, Cohen Children’s Medical Center, Northwell Health, New Hyde Park, NY USA; 22 Division of Pediatric Surgery, Joe Di Maggio Children’s Hospital, Memorial Healthcare System, Hollywood, FL USA

**Keywords:** Interval appendectomy, Complicated appendicitis, Non-operative management, Pediatric

## Abstract

**Background:**

This review summarizes considerations within the existing recent literature that guide the practice of interval appendectomy (IA) after initial non-operative management (NOM) of complicated appendicitis (CA) in children.

**Methods:**

A systematic review of English language articles published from 2000 to 2025 was conducted in Medline, Embase, and Cochrane Central Register of Controlled Trials to address four elements which could impact the decision for IA after NOM of CA: (1) the incidence of recurrent appendicitis; (2) the time period in which recurrence occurs; (3) the patient or disease-related risk factors which increase recurrence; and (4) the incidence of appendiceal neoplasms identified by IA.

**Results:**

Of the 3,022 articles initially reviewed, 46 met inclusion criteria. Recurrence was reported in 2–50% of patients. When IA is pursued, the optimal timing remains undefined, although evidence suggests most recurrences occur within three to six months, so there may be potential benefit to performing IA within three months after the initial presentation. Risk factors for recurrent appendicitis are not well characterized, though the presence of an appendicolith may increase recurrence risk. Across studies, the incidence of appendiceal neoplasms was rare, with most studies not documenting any cases; all reported neoplasms were neuroendocrine tumors.

**Conclusion:**

Current evidence does not favor any single approach to IA, including routine IA, selective IA, or indefinite NOM; rather a shared-decision ought to be made between the surgeon and patient’s caregivers. Utilization and timing of IA must balance surgical risks with the risk of recurrent disease; however, the risk of neoplasm should not primarily drive management.

**Level of Evidence:**

I−IIV.

**Supplementary Information:**

The online version contains supplementary material available at 10.1007/s00383-026-06445-z.

## **Introduction**

Appendicitis is the most common indication for urgent abdominal surgical intervention in the pediatric population. Of these, nearly 30% of children present with perforation or advanced disease, also referred to as complicated appendicitis (CA) [1, 2]. Urgent surgery was once the standard of care for all patients with appendicitis. Advances in broad-spectrum antibiotics and interventional radiology techniques have expanded management options and select cases of CA may now be treated non-operatively at the initial, index admission [3]. Recently, the Society of American Gastrointestinal and Endoscopic Surgeons published guidelines on appendicitis management that recommended routine interval appendectomies in children based only on expert opinion, as the low-quality of evidence did not allow them to make an evidence-based recommendation [4]. There remains limited high quality data to inform practice and little consensus on best practices following successful non-operative management (NOM) of CA, specifically as it relates to consideration for interval appendectomy (IA).

Appendectomy following successful NOM of CA remains controversial, and in those who pursue IA, the optimal timing of surgery has not been well-defined. The decision to perform an IA should weigh the risks of recurrent appendicitis and/or missed neoplasm against the potential risks of anesthesia and perioperative complications. Additionally, costs, quality of life and other factors such as disability days may guide practice. Therefore, the Outcomes and Evidence-Based Practice (OEBP) committee of the American Pediatric Surgical Association (APSA) performed a systematic review to investigate some of the considerations that drive the practice of IA following NOM for CA.

## Methods

### Research questions

Through group consensus, the APSA OEBP committee composed the following questions to perform this systematic review:

1. What is the incidence of recurrent appendicitis following initial non-operative management of complicated appendicitis?
2. What is the timing of recurrent appendicitis following initial non-operative management of complicated appendicitis?
3. What are the risk factors for recurrent appendicitis following initial non-operative management of complicated appendicitis?
4. How often are incidental neoplasms (neuroendocrine tumors, carcinoma, etc.) identified during interval appendectomy?

### Search methods and data sources

The study protocol was registered with PROSPERO and approved in December 2023 (CRD42023485154). Queries of the publicly available databases Medline (Ovid) 1946–2024, Embase (Elsevier) 1974–2024, and the Cochrane Central Register of Controlled Trials (Wiley) 2004–2024 were conducted in February 2024. An additional search was performed to identify additional studies published during 2024 and 2025. A health sciences librarian developed the search strategy for the primary database (Medline) using a combination of keyword searching and database index Medical Subjects Heading (MeSH) terms (Supplementary Table 1). The primary search was peer-reviewed by a second health sciences librarian according to Peer Review of Electronic Search Strategies (PRESS) guidelines [5]. After addressing peer review recommendations and comments, the primary database strategy was translated for use in additional databases. The Embase search filtered out results indexed as conference abstracts.

### Study selection

Studies included in this systematic review were published between January 1, 2000 and December 31, 2025 and reported on pediatric (younger than 18 years of age) patients with CA who underwent successful NOM during their initial hospital stay (index admission). Excluded studies (*n* = 2,971) involved: those with adult patients only (18 years old or older), those with both adult and pediatric patients but subgroup analysis of pediatric patients (younger than 18 years old) was not conducted, those for whom analysis of CA cases was not conducted, case reports or series analyzing fewer than three pediatric patients, those utilizing an administrative database, those that did not address any outcome measure associated with the study questions, review manuscripts, opinion pieces, those written in a language other than English without an accompanying English language translation, and those outside of the study timeframe. Studies published prior to the year 2000 were excluded as this represented a time when laparoscopic appendectomy became the predominant surgical technique [6]. The Preferred Reporting Items for Systematic Reviews and Meta-Analyses (PRISMA) were used to organize this study [7]. Studies were reviewed using Covidence (covidence.org), with two unique authors reviewing each abstract at the initial stage of review and then again at the full manuscript stage; conflicts were adjudicated by a third author.

### Definitions

Given wide variability in terminology noted across studies, several definitions were agreed upon to ensure uniformity when reviewing the literature. *Complicated appendicitis* was an inclusion criterion decided by the authors of each included paper, however without any known exception this referred to the widely used definition of the term as representing visible perforation or advanced disease with abscess, phlegmon, extraluminal fecalith or diffuse generalized peritonitis [8]. Although strictly speaking an *interval appendectomy* is an elective procedure, that is not how many studies defined the term, instead including both elective and urgent appendectomies under the same term. For this reason, IA was considered *planned* if the a priori decision was to perform an IA and this was done in an elective fashion, and was considered *unplanned* if the patient underwent an urgent or emergent appendectomy for any indication including recurrent appendicitis, persistent symptoms, or change in patient/parental choice. *Recurrent appendicitis* was defined as requiring another course of treatment of appendicitis (medical or surgical) after being successfully discharged following NOM for CA.

### Data extraction and analysis

The included manuscripts were critically appraised utilizing median methodological items for non-randomized studies criteria and were assigned a level of evidence (LOE) as per the methodological criteria from the Oxford Centre for Evidence-Based Medicine [9].

Outcomes of interest included the total number of patients with successful NOM at index admission, the number of patients who underwent IA after successful NOM subcategorized as planned or unplanned, the incidence of recurrence before scheduled IA or during the observation period, the timing of planned and unplanned IA, timing of recurrences, risk factors for recurrence, and the incidence of neoplasms noted during IA. The number of occurrences was collected for each individual outcome of interest. Percentages were then calculated based on these numbers within each study. Ranges of percentages were determined for each outcome as appropriate.

A consensus statement for each recommendation was generated in response to each research query by using the Grading of Recommendations, Assessment, Development, and Evaluation (GRADE) criteria and allocating a grade between A-D.

## Results

### Overview of studies

**Fig. 1:**
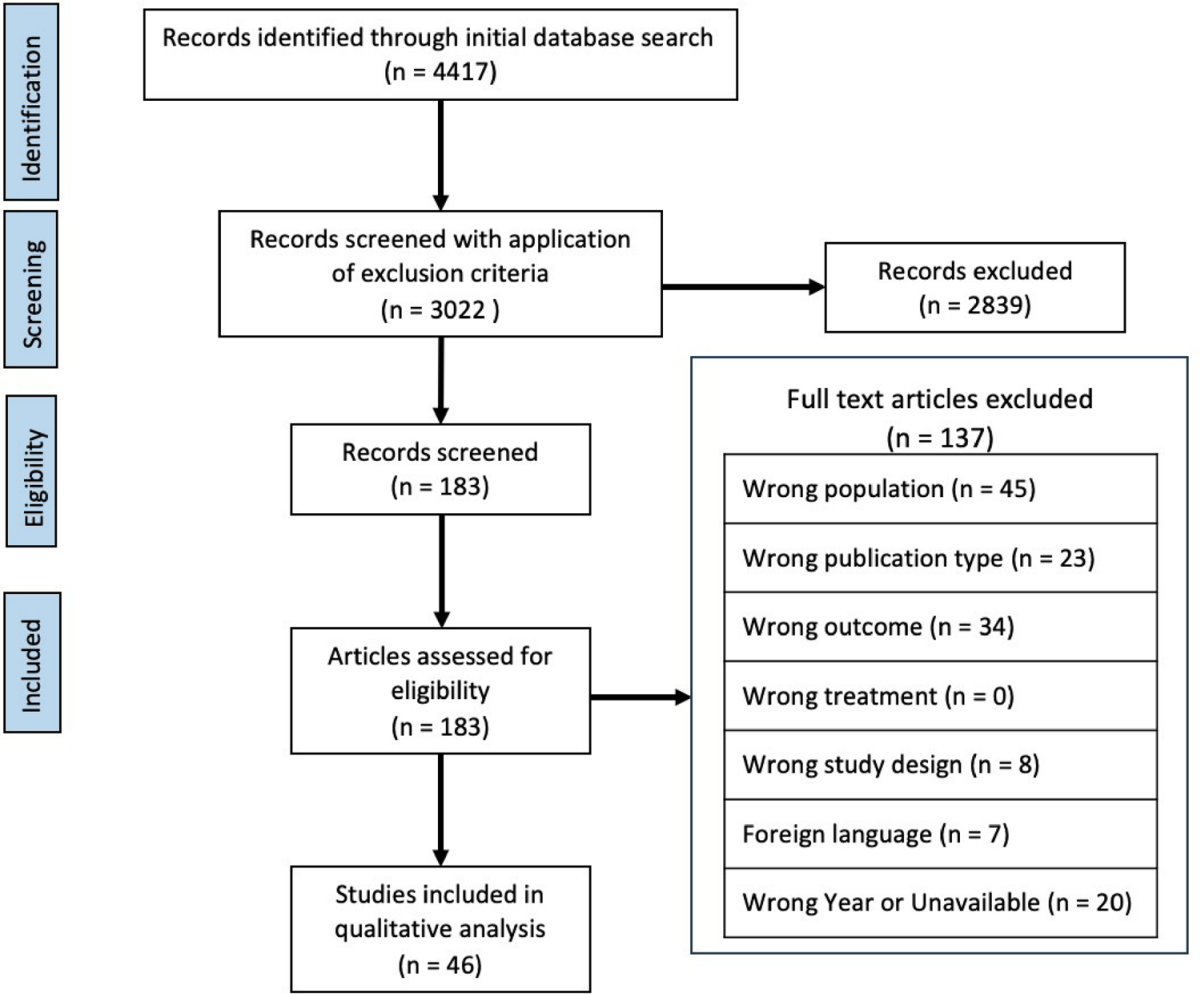
Diagram of study selection according to the Preferred Reporting Items for Systematic Reviews and Meta-Analyses (PRISMA) criteria

The initial query resulted in 4,416 abstracts. One additional abstract was found through citation review, for a total of 4,417 abstracts to review. There were 1,395 duplicate abstracts that were excluded. The remaining 3,022 underwent initial abstract review and 183 studies received full manuscript analysis. Forty-six studies satisfied criteria to be included in the final literature review (Fig. 1). These 46 studies described 6,222 combined patients. Thirty six studies were retrospective [10–45], three studies were randomized controlled trials [46–48], and the remaining seven were prospective observational studies [49–55]. Four studies did not include any patients who underwent a planned IA [14, 28, 43, 54] (Ein et al. [14] excluded the 41 patients who underwent a planned IA and focused their analysis on the remaining 49), five explicitly included only patients who had a planned IA [10, 11, 25, 27, 39], and the remainder (37) included patients who had a planned IA, unplanned IA, or continued NOM. Table 1 includes a summary of the number of patients in each study who underwent an appendectomy and whether it was planned or unplanned. As an indicator of variability in practices across studies, 0% to 100% of all patients who were successfully discharged after NOM of CA were reported as having undergone a subsequent appendectomy (planned or unplanned).

**Table 1:** Incidences of total, planned, unplanned interval appendectomies (IA) and recurrent appendicitis in included studies, among patients who had successful non-operative management (NOM) for complicated appendicitis

Study	Successful NOM (*n*)	Total IA, *n* (%)	Planned IA, *n* (%)	Unplanned IA, *n* (%)	Recurrence, *n* (%)
Aprahamian CJ, et al. 2007 [45]	75	75 (100)	66 (88)	9 (12)	9 (12)
Badru F, et al. 2019 [42]	148	148 (100)	143 (96.6)	5 (3.4)	5 (3.4)
Blakely ML, et al. 2011 [48]	67	65 (97)	42 (62.7)	23 (34.3)	6 (9.0)
Bass, et al. 2006 [10]	32	32 (100)	32 (100)	n/a	n/a
Calvert CE, et al. 2014 [11]	64	58 (90.6)	58 (90.6)	0	0
Castello Gonzalez M, et al. 2014 [12]	41	20 (48.8)	16 (39)	4 (9.8)	4 (9.8)
Church JT, et al. 2017 [13]	55	54 (98.2)	49 (89.1)	5 (9.1)	5 (9.1)
Ein SH, et al. 2005 [14]	90	62 (68.9)	41 (45.6)	21 (23.3)	21 (23.3)
Emil S, Duong S. 2007 [15]	28	28 (100)	26 (92.9)	2 (7.1)	2 (7.1)
Erdogan D, et al. 2005 [16]	21	21 (100)	19 (90.5)	2 (9.5)	2 (9.5)
Farr BJ, et al. 2021 [17]	510	510 (100)	500 (98.0)	10 (2.0)	10 (2.0)
Fawkner-Corbett D, et al. 2014 [18]	69	69 (100)	61 (88.4)	8 (11.5)	8 (11.5)
Fawley J, Gollin G. 2013 [19]	212	212 (100)	185 (87.3)	27 (12.7)	27 (12.7)
Fouad D, et al. 2020 [20]	149	149 (100)	143 (96.0)	6 (4.0)	36 (24.2)
Furuya T, et al. 2015 [21]	16	16 (100)	14 (87.5)	2 (12.5)	2 (12.5)
Gahukamble DB, et al. 2000 [22]	54	39 (72.2)	32 (59.3)	7 (17.9)	7 (17.9)
Ghidini F, et al. 2023 [41]	26	26 (100)	23 (88.5)	3 (11.5)	3 (11.5)
Gillick J, et al. 2008 [24]	97	97 (100)	97 (100)	n/a	n/a
Gillick J, et al. 2001 [23]	349	349 (100)	331 (94.8)	18 (5.2)	21 (6.0)
Hall NJ, et al. 2017 [47]	102	56 (54.9)	44 (43.1)	12 (11.7)	12 (11.7)
Iqbal CW, et al. 2012 [25]	128	128 (100)	128 (100)	n/a	n/a
James IA, et al. 2011 [44]	117	116 (99.1)	94 (80.3)	22 (18.8)	22 (18.8)
Kim E, et al. 2021 [26]	43	43 (100)	43 (100)	0 (0)	0 (0)
Luo CC, et al. 2016 [27]	1225	263 (21.5)	185 (15.1)	78 (6.4)	78 (6.4)
Luo PC, et al. 2022 [28]	124	0 (0)	0 (0)	0 (0)	62 (50)
Munoz A, et al. 2019 [49]	69	69 (100)	51 (73.9)	18 (26.1)	18 (26.1)
Nadler E, et al. 2004 [29]	68	68 (100)	54 (79.4)	14 (20.6)	14 (20.6)
Nazarey PP, et al. 2014 [50]	105	105 (100)	93 (88.6)	12 (11.4)	36 (34.3)
Otake S, et al. 2014 [30]	48	43 (89.6)	43 (89.6)	0	5 (10.4)
Owen A, et al. 2006 [31]	36	36 (100)	35 (97.2)	1 (2.8)	1 (2.8)
Parmentier B, et al. 2016 [51]	64	58 (90.6)	54 (84.4)	4 (6.3)	10 (15.6)
Pederiva F, et al. 2021 [32]	40	39 (97.5)	33 (82.5)	6 (15)	6 (15)
Pennell C, et al. 2021 [52]	58	57 (98.3)	53 (91.4)	4 (4.1)	4 (4.1)
Puapong D, et al. 2007 [33]	72	16 (22.2)	11 (15.3)	5 (6.9)	5 (6.9)
Roach JP, et al. 2007 [34]	32	32 (100)	32 (100)	0 (0)	0 (0)
Saluja S, et al. 2018 [35]	848	848 (100)	597 (70.4)	251 (29.6)	251 (29.6)
Samuel M, et al. 2002 [53]	48	48 (100)	29 (60.4)	19 (39.6)	19 (39.6)
St. Peter SD, et al. 2010 [46]	18	18 (100)	16 (88.9)	2 (11.1)	2 (11.1)
Svensson JF, et al. 2014 [54]	89	9 (10.1)	0 (0)	9 (10.1)	12 (13.5)
Takahashi Y, et al. 2021 [36]	34	29 (85.3)	29 (85.3)	0 (0)	5 (14.7)
Talishinskiy T, et al. 2016 [37]	58	58 (100)	52 (89.7)	6 (10.3)	6 (10.3)
Tanaka Y, et al. 2016 [55]	55	29 (52.7)	15 (27.3)	12 (21.8)	14 (25.5)
Vane DW, Fernandez N. 2006 [39]	27	27 (100)	27 (100)	0 (0)	0 (0)
Whyte C, et al. 2008 [39]	37	31 (83.8)	31 (83.8)	n/a	n/a
Zhang HL, et al. 2013 [43]	103	5 (4.9)	0 (0)	5 (4.9)	14 (13.6)
Zhang Y, et al. 2021 [40]	471	471 (100)	385 (81.7)	86 (18.3)	86 (18.3)

Percentages here are out of the total number of patients in each study who underwent successful NOM, so may differ from how it is reported elsewhere

When planned, timing of elective IAs varied widely with surgery scheduled for as short as 4 weeks (631 patients) [23, 24, 39, 42], 6–8 weeks (505 patients) [11, 20, 22, 29, 31, 34, 38, 45], 8–12 weeks (602 patients) [14, 16, 19, 32, 37, 46, 50, 52], or greater than 12 weeks (1070 patients) [17, 36, 40, 55] following index admission. Recommended timing of elective IA, when described by the authors of some papers, was frequently based on the perceived timing of recurrence balanced with operative complexity secondary to ongoing inflammation. Although one group identified greater odds of histologic acute inflammation prior to 12 weeks from index admission [17], several other groups have demonstrated ongoing inflammation of the appendix histologically throughout the first 6 months with no correlation between time from diagnosis and degree of inflammation [10, 30, 32, 39].

### Question What is the incidence of recurrent appendicitis following initial non-operative management of complicated appendicitis?

Forty-one studies addressed Question 1 and included a total of 4736 patients. Of these, four studies included no patients who underwent a planned, elective IA, opting instead for observation [14, 28, 43, 54]. The remaining 37 studies included a mix of patients who underwent either a planned, elective IA; an unplanned, urgent IA due to recurrence; or no appendectomy [12–23, 26, 27, 29–33, 35–42, 44, 45, 47–53, 55]. All together, these studies reported recurrence rates ranging from 2.0% to 50%. Recurrence was identified in 772 patients (16.3%) who were managed medically or surgically. Unplanned IA was performed in 640 (13.5%) for a variety of indications including recurrence and parent preference, however 3393 (71.6%) underwent a planned IA which excluded them from being at risk for recurrence.

There were four studies where no planned IAs were performed; in these the recurrence rates were 12 out of 89 (13.5%), 14 out of 103 (13.6%), 21 out of 90 (23.3%), and 62 out of 124 (50%) [14, 28, 43, 54]. Also, the one randomized controlled trial that directly compared observation to planned IA reported that the observation group had six cases of histology-proven appendicitis but an additional six patients in this group underwent an appendectomy for other related indications; thus the recurrence rate as defined here is 12 out of 52, or 23% [47] .

Among the other 37 studies that included planned IAs in the analysis, between 15.1% and 100% of patients ultimately underwent a planned IA, while an unplanned IA occurred in up to 39.6% of the patients.

*Summary of Question 1*. Overall recurrence rates ranged from 2.0% to 50% and 13.5% to 50% among studies that only included unplanned IA.

*Recommendation*. Data regarding the incidence of recurrence after NOM for complicated appendicitis are not sufficient to specifically recommend performing IA routinely or only in cases of recurrent appendicitis; instead, a shared decision between the surgeon and the patient and their family should be made balancing the risks and benefits of either approach.

Grade D recommendation.

### Question What is the timing of recurrent appendicitis following initial non-operative management of complicated appendicitis?

There was wide variability in how the time to recurrence was measured and documented across studies, making pooled analysis unfeasible. Most studies reported recurrences occurring within three months of the index admission (92 recurrences out of 1061 patients) [17, 18, 21, 22, 33, 36, 45, 46, 49, 54, 55]. Some identified recurrences up to six months after the index admission (36 recurrences out of 703 patients) [17, 21, 22, 36, 54]. Other studies reported on recurrences beyond six months after the index admission (81 recurrences out of 486 patients), suggesting that the risk of recurrence remains as long as the appendix remains intact [14, 16, 31–33, 43, 49, 55]. Reported averages and medians of time to recurrent appendicitis ranged between 1 and 37 weeks.

As an example, Gahukamble et al. simply reported that the seven recurrences occurred between 15 days and 6 months from the index admission [22]. Svensson et al. reported that the two recurrences were identified at 122 and 123 days post-discharge [54, 14, 43]. An additional study worth noting for the length of follow up is that by Puapong et al., who followed 61 patients for a mean of 7.5 years (range 2 months − 12 years). They identified five (8%) patients who developed recurrent appendicitis all within 3 years of initial diagnosis and 80% within 6 months of index hospitalization [33].

*Summary of Question 2*. Most cases of recurrent appendicitis occurred within three to six months of the index admission with continued risk of recurrence beyond this period.

*Recommendation*. Based on limited data available, if an IA is pursued, timing it three months from index admission may be considered. Regardless of the management plan, families should be counseled about the likely greater risk of recurrent appendicitis within three to six months after the index admission.

Grade D recommendation.

### Question What are the risk factors for recurrent appendicitis following initial non-operative management of complicated appendicitis?

Thirteen studies encompassing 1338 patients were identified that evaluated risk factors for recurrent appendicitis [14, 15, 20, 23, 28, 36, 41, 43, 44, 49, 50, 54, 55]. Two of these studies were prospective, non-randomized studies [50, 55], and the remainder were retrospective studies [14, 15, 20, 23, 28, 36, 41, 43, 44, 49, 54]. The percentage of patients across these studies who underwent an unplanned IA, due to recurrent appendicitis or another indication (e.g., parental choice, persistent symptoms), ranged from 5.2% to 50%. Further details are summarized in Table 2.

**Table 2:** Risk factors and indications noted by studies to be related to an unplanned interval appendectomy (IA). Percentages given for each variable are the rate of recurrence or unplanned IA

Study	Recurrence%	Appendicolith	Elevated WBC	Small bowel obstruction	Pain duration	Other
Ein SH, et al. 2005 [14]	42.8%	72% with vs. 26% without				
Emil S, Duong S. 2007 [15]	7.1%					Phlegmon on initial imaging, (4 out of 4 recurrences)
Fouad D, et al. 2020 [20]	24.2%	32% with vs. 23% without				
Ghidini F, et al. 2023 [41]	11.5%	25% of recurrences with				
Gillick J, et al. 2001 [23]	6%	67% of unplanned IA with vs. 10% of planned IA with		6% with		
James I, et al. 2011 [44]	18.8%	**45.5% with vs. 14.9% without**				
Luo P et al. 2022 [28]	50%		**WBC > 8**,**000/mL OR 2.7 (95% CI 1.2–6.2)**			**Ratio of abscess size to BSA > 4.3 OR 1.4 (95% CI 1.1–1.7)**
Munoz A, et al. 2019 [49]	26.1%		**14**,**900/mL vs. 18**,**900/mL**	25% vs. 0%	**2.8 days vs. 4.1 days**	**Age 7.5 (SD: 2–14) years vs. 11 (2–16) years**
Nazarey PP, et al. 2014 [50]	34.3%	27.2% with vs. 7.8% without*	**WBC > 15**,**000/mL 23.7% vs. 4.5%**		28.6% >48 h vs. 8.8% without*	
Svensson JF, et al. 2014 [54]	13.5%	~ 50% with vs. 46% without**				Parent choice
Takahashi Y, et al. 2021 [36]	14.7%					
Tanaka Y, et al. 2016 [55]	25.5%		11,700/mL vs. 15,200/mL			**Temperature 37.1 C vs. 38.2 C**
Zhang HL, et al. 2013 [43]	13.6%	19.1% with vs. 8.9% without				

NB: When not otherwise specified, the first value given is the result for the group that had a recurrence followed by the result for the group that did not. Bold denotes a statistically significant (*p* < 0.05) result. * denotes in this study this variable was combined with WBC > 15,000/mL. ** denotes an estimated value based on interpretation of the text and not a reported result. Abbreviations: WBC, white blood cell; OR, odds ratio; 95% CI, 95% confidence interval; BSA, body surface area; SD, standard deviation

The risk factor most commonly described as a predictor for recurrence was the presence of an appendicolith. Nazarey et al., Ein et al., and James et al. all found that presence of a fecalith was more likely to result in recurrence and unplanned IA (respectively: 27.2% vs. 7.8%, *p* = 0.05; 72% vs. 26%, *p* = 0.004; 45.5% vs. 14.9%, *p* = 0.003) [14, 44, 50, 50]. Of the 33 appendicoliths identified on pathology by Gillick et al., most (29, 87.9%) were found in cases of unplanned IA [23]. Zhang et al. did not find a significant difference in recurrence for patients with or without appendicoliths (19.1% vs. 8.9%, *p* = 0.132) ; but on subsequent cross-sectional imaging (CT scans) found that 38 of the 49 patients had disappearance of the appendicolith with recurrence 7.9% in the group with resolution compared with 66.7% in the group with a persistent appendicolith (*p* < 0.05) [43].

On the other hand, some studies failed to support the association of appendicoliths with recurrent appendicitis [20, 28, 36, 41, 43, 49, 54, 55]. Of the two recurrences that Svensson et al. identified, one had an appendicolith found (50%) and the other did not. In their study, 41 of the total 89 patients (46%) had appendicoliths, and they were unable to report that this was predictive of failure [54]. Similarly, Ghidini et al. and Tanaka et al. found no difference in recurrence based on presence of an appendicolith [41, 55]. Luo et al. and Fouad et al. found a higher rate of appendicoliths on imaging and pathology respectively, however this did not statistically significantly increase incidence of recurrent appendicitis (respectively: 37.1% vs. 22.6%, *p* = 0.077; 32% vs. 23%, *p* = 0.316) [20, 28].

Some studies did identify factors other than appendicolith which may impact recurrence. Munoz et al. identified younger age and lower WBC as risk factors of recurrence [49]. Emil et al. demonstrated that those patients who failed NOM had a phlegmon on their initial imaging [15]. Finally, Luo et al. found that the ratio of the abscess size to body surface area was significantly associated with development of recurrence (*p* < 0.001) [28]. Notably, some studies, including Tanaka et al. and Takahashi et al. did not find any predictors of recurrence in their analysis [36, 55].

*Summary of Question 3*. Overall reporting of risk factors for recurrent appendicitis was inconsistent but the presence of an appendicolith may be associated with an increased risk of recurrent appendicitis.

*Recommendation*. The presence of an appendicolith on imaging may be associated with an increased risk of recurrence and therefore is a reasonable consideration for those selectively recommending an IA.

Grade D recommendation.

### Question How often are incidental neoplasms (neuroendocrine tumors, carcinoma, etc.) identified during interval appendectomy?

Seventeen studies reported on appendix pathology following IA [10, 15–18, 20, 23, 25, 30–32, 39, 41, 47, 50, 53, 54]. In total, these studies included 1,757 patients who underwent IA (Table 3), with only five studies (974 patients) identifying a total of 12 cases with neoplasms [17, 23, 31, 39, 53] and the rest reporting no neoplasms. Each of the five studies reported between one and six cases of neoplasm, accounting for study-specific incidences of 0.6–5.6%. All identified tumors were neuroendocrine tumors; no cases of carcinoma were identified. All the tumors identified in these studies were managed by simple appendectomy.

**Table 3:** Incidence of neoplasm for patients undergoing interval appendectomy (IA) following non-operative management of complicated appendicitis

Study	Total IA (*n*)	Total planned IA (*n*)	Total unplanned IA (*n*)	Total IA with neoplasm (*n*)*	Total IA with neoplasm (%)
Bass, J, et al. [10]	32	32	0	0	0
Emil S, et al. [15]	28	26	2	0	0
Erdogan D. et al. [16]	21	19	2	0	0
Farr B, et al. [17]	510	500	10	6	1.2
Fawkner-Corbett D, et al. [18]	69	61	8	0	0
Fouad D, et al. [20]	149	143	6	0	0
Ghidini F, et al. [41]	24	21	3	0	0
Gillick J, et al. [23]	349	331	18	2	0.6
Hall N, et al. [47]	56	44	12	0	0
Iqbal C, et al. [25]	128	128	0	0	0
Nazarey P, et al. [50]	105	93	12	0	0
Otake S, et al. [30]	43	43	0	0	0
Owen A, et al. [31]	36	35	1	2	5.6
Pederiva F, et al. [32]	39	33	6	0	0
Samuel M, et al. [53]	48	29	19	1	2.1
Svensson J, et al. 2014 [54]	89	0	9	0	0
Whyte C, et al. [39]	31	31	0	1	3.2

* All neoplasms were present in specimens obtained during planned IA with exception of Samuel M, et al. which was not specified

*Summary of Question 4*. The incidences of neoplasms at the time of IA following NOM of CA ranged between 0 and 5.6%, but only five studies reported any such cases while 12 studies reported no cases at all. All cases were neuroendocrine tumors and all were removed with appendectomy alone.

*Recommendation*. Routine IA is not necessary for prophylactic removal of a potential appendiceal neoplasm given the low incidence, although counseling families about this risk is important.

Grade D recommendation.

## Discussion

This APSA OEBPC review highlights the current controversies in existing literature and variations in practice for the subsequent management of pediatric patients who successfully undergo upfront non-surgical management of CA. We sought to evaluate the available evidence regarding the decision and timing for IA if pursued including identifying those patients most likely to benefit.

Across all studies included in our review, the rate of recurrent appendicitis and unplanned IA following NOM for CA ranged from 2 to 50%, and was between 13.5% and 50% in those studies where no planned IAs were performed. The amount of time to recurrence also ranged widely, from just several days to several years after index admission, but most occurred within three to six months with additional recurrences identified when followed for longer. Data regarding risk factors for recurrent appendicitis were not reported in a consistent or uniform way across studies, limiting generalizability and our ability to draw any evidence-based conclusions. The most cited predictor of failure of long-term NOM was presence of an appendicolith; however this result was inconsistent across studies. Several studies mentioned repeat imaging studies to assess for persistent appendicolith, but this practice and its utility was outside the scope of this review. Finally, the overall incidence of neoplasms following CA was found to be low, ranging from 0 to 5.6% across the studies reporting pathology, but of the 17 studies evaluating pathology only five reported a malignancy and this represented only 12 out of 1,757 total patients across those studies.

While there are numerous manuscripts detailing the NOM of CA, there are few well-controlled studies. The body of literature is heterogeneous, with various study designs, differing populations, and variables with inconsistent definitions being collected and analyzed. Among the included manuscripts comparing IA to observation following resolution of symptoms after index admission, the majority were retrospective series in which reported outcomes often reflected preferred management paradigms with inherent biases and only one was a prospective randomized controlled trial (RCT) comparing planned IA to observation after NOM. This RCT highlights how the variations in terminology impacted how results were reported and interpreted: the authors reported a recurrence rate of six out of 52 in the observation arm (11.5%) based on histologic findings, however six additional patients ultimately underwent an appendectomy in the observation group, resulting in overall incidence of 23% of patients undergoing an appendectomy [47]. When counseling parents, the histology-confirmed recurrence rate is less useful than how many patients ultimately undergo an unplanned IA after NOM.

Also of note, a recent study utilizing the Pediatric Health Information System (PHIS) administrative database (not included in overall analysis given that it did not meet inclusion criteria) found that of 2,826 patients who were treated non-operatively for CA 810 (28.7%) did not have a documented subsequent appendectomy and 332 (11.7%) had a subsequent unplanned readmission presumably for recurrent appendicitis [56]. The authors also reported that most unplanned readmissions occurred within 50 days of the index admission, suggesting that most recurrences happen early. The authors proposed that these findings can help surgeons determine the timing of interval appendectomies and may support parents who wish to pursue observation rather than surgery.

Data regarding the risk of recurrence are particularly difficult to interpret, since the majority of studies included at least some patients who underwent a planned IA. By removing the appendix a short time after the index admission for CA, those patients cannot any longer be considered at risk for developing recurrence so the denominator for the risk calculation continues to decline, leading to potential overestimation of true risk of recurrence. For this reason we highlighted the recurrence outcomes from studies that included patients managed with observation. This fact, along with additional heterogeneity in how variables were defined, led to the determination that it was not statistically sound to pool data and perform a meta-analysis.

Two important outcomes that were not included in this systematic review are surgical complications of IA and cost. Peri-operative complications were frequently not addressed and when included were done so in a way that did not allow for aggregation or comparison across studies. When reported, most were superficial or deep surgical site infections [14, 18, 20, 23, 25, 26, 41].

With respect to cost, there are studies that have compared the costs of early upfront appendectomy for CA versus NOM followed by IA [13, 26, 38, 46, 57]. These have shown a cost advantage for observation versus planned IA [47] and for planned IA when compared to unplanned IA [18]. Raval et al. developed a model based on published outcomes to investigate this question and found a cost advantage for foregoing routine IA [58] thereby supporting observation when considering costs alone. Observation may be less costly on average than routine IA, but it is important to discuss with families that an unplanned IA may ultimately be more costly *for them* than a planned IA. Complications and cost, plus other patient-centered outcomes such as quality of life indicators and time away from school, work or activities, should be considered for future research on this topic.

Focusing on patient-centered outcomes including length of stay, disability days, and patient satisfaction, as well as complications and costs would ensure that future studies identify the optimal choice with respect to the patient’s experience as well as what is most appropriate from a surgical perspective. Designing a well-powered, prospective study would better address these open questions to aid in evidence-based decisions and recommendations.

Unique limitations in our study had to do with varied and inconsistent definitions of study terms. Most importantly, we were unable to perform a meta-analysis or a more basic pooled analysis of any outcomes given the data available and heterogeneity of results. A cohort that included planned IAs (recommended for all patients or selectively) will fundamentally be different than another where they were not, so outcomes could not appropriately be combined or compared. Also, the definition of CA has evolved in the literature and there may have been some variation from one study to another for this inclusion criterion. Additionally, recurrent appendicitis was defined by some groups as occurring days after discharge from index admission, whereas some may consider this persistent disease rather than a true recurrence. Authors may have referred to recurrent appendicitis without specifying an intervening period of symptom resolution. Duration of antibiotics, differing antibiotic regimens, and indications for drainage of abscesses may impact the risk of recurrence and oftentimes were not specified or standardized. Timing to planned IA differed among groups, with resultant limitations in determining true risk of recurrence and timing to recurrence as early appendectomy limits patients eligible for long term follow-up. Finally, regarding risk of neoplasm, it is important to note that studies included in this review only considered pathology among patients with CA who underwent IA and did not include patients with simple appendicitis, those with CA treated with upfront appendectomy, or those treated with medical management alone. Given the large population excluded, this ought not be considered an authoritative determination of the neoplasm risk in pediatric appendicitis overall.

## Conclusions

The incidence of recurrent appendicitis following initial successful NOM of CA ranged from 2 to 50% with the rate among studies with no planned IAs 13.5% to 50%. Most typically, recurrence occurred within three to six months of the index admission indicating that if pursued, a planned IA may be considered within three months with counseling that the risk of recurrent appendicitis remains beyond this time period. The presence of an appendicolith may be predictive of failed long-term NOM, but this was not consistent across all studies. The neoplasm risk was low; most studies found no neoplasms and those that were identified were all benign neuroendocrine tumors managed by appendectomy alone. While these data may help surgeons with future shared decision-making, they do not support any single practice pattern. Further research, focusing on patient-centered outcomes in particular is warranted.

## Supplementary Information

Below is the link to the electronic supplementary material.

Supplementary file1

## Data Availability

No datasets were generated or analysed during the current study.
