# Supplementary material for: Interval appendectomy practices for complicated appendicitis in children: a systematic review from the APSA Outcomes and Evidence-Based Practice Committee
Source: Pediatr Surg Int. 2026 Apr 27;42(1):205. doi: 10.1007/s00383-026-06445-z (PMC13121297; doi:10.1007/s00383-026-06445-z)
Supplement: Supplementary file 1 — Supplementary file1 [file 383_2026_6445_MOESM1_ESM.docx]

Supplementary Table 1: Medical Subject Headings Query Terms

| **Term Category** | **MeSH Query Term** |
| --- | --- |
| Appendicitis | recurrent appendicitis; complicated appendicitis; perforated appendicitis; advanced appendicitis; append (British) |
|  |  |
| Pediatric | Infant*; newborn*; new-born*; perinat*; neonat*; baby; baby*; babies; toddler*; minors; minors*; boy; boys; boyfriend; boyhood; girl*; kid; kids; child; child*; children*; schoolchild*; schoolchild; school child [tiab]; school child*; adolescen* ; juvenil*; youth*; teen*; under*age*; pubescen*; pediatrics[mh]; pediatric*; paediatric*; peadiatric*; school; school*[tiab]; prematur*; preterm* |
| Surgery | interval appendectomy; delayed appendectomy; non-operative management appendicitis |
